# Slow and Fast Responses: Two Mechanisms of Trial Outcome Processing Revealed by EEG Oscillations

**DOI:** 10.3389/fnhum.2017.00218

**Published:** 2017-05-05

**Authors:** Nikita A. Novikov, Yulia M. Nurislamova, Natalia A. Zhozhikashvili, Evgenii E. Kalenkovich, Anna A. Lapina, Boris V. Chernyshev

**Affiliations:** ^1^Laboratory of Cognitive Psychophysiology, National Research University—Higher School of EconomicsMoscow, Russia; ^2^Department of Higher Nervous Activity, Lomonosov Moscow State UniversityMoscow, Russia

**Keywords:** cognitive control, attention, response time, error detection, theta oscillations, alpha oscillations, beta oscillations

## Abstract

Cognitive control includes maintenance of task-specific processes related to attention, and non-specific regulation of motor threshold. Depending upon the nature of the behavioral tasks, these mechanisms may predispose to different kinds of errors, with either increased or decreased response time (RT) of erroneous responses relative to correct responses. Specifically, slow responses are related to attentional lapses and decision uncertainty, these conditions tending to delay RTs of both erroneous and correct responses. Here we studied if RT may be a valid approximation distinguishing trials with high and low levels of sustained attention and decision uncertainty. We analyzed response-related and feedback-related modulations in theta, alpha and beta band activity in the auditory version of the two-choice condensation task, which is highly demanding for sustained attention while involves no inhibition of prepotent responses. Depending upon response speed and accuracy, trials were divided into slow correct, slow erroneous, fast correct and fast erroneous. We found that error-related frontal midline theta (FMT) was present only on fast erroneous trials. The feedback-related FMT was equally strong on slow erroneous and fast erroneous trials. Late post-response posterior alpha suppression was stronger on erroneous slow trials. Feedback-related frontal beta was present only on slow correct trials. The data obtained cumulatively suggests that RT allows distinguishing the two types of trials, with fast trials related to higher levels of attention and low uncertainty, and slow trials related to lower levels of attention and higher uncertainty.

## Introduction

Cognitive control is a functional set of processes that provides maintenance of adaptive goal-directed behavior (Botvinick et al., 2001; Yeung, 2014). In perceptual decision tasks, high performance depends on successful encoding of sensory information, followed by activation of stimulus-to-response mapping representation, ultimately leading to appropriate response selection. In such tasks, cognitive control is responsible for maintenance of task-specific processes related to sustained attention (which enhances sensory encoding and activation of appropriate stimulus-to-response mappings), as well as for non-specific regulation of motor threshold (which increases the chances that correct motor programs will win the competition against incorrect ones; Ridderinkhof, 2002; Dudschig and Jentzsch, 2009; King et al., 2010; Danielmeier and Ullsperger, 2011; Cohen, 2014). In line with this distinction, performance errors may have two different mechanisms: some errors may result from inappropriate action impulses that were not inhibited due to lowered motor threshold, while others may result from lapses in sustained attention that deteriorate stimulus encoding and subsequent activation of stimulus-to-response mapping (van Driel et al., 2012). Response time (RT) of erroneous responses tends to be shorter than correct RT in the first case (“error speeding”) and longer—in the second case (“error slowing”).

The nature of the task may predispose to one or the other error type, with either “error speeding”, or “error slowing”, respectively. Erroneous RT tends to be shorter if a task is simple and/or it requires fast rather than accurate responses (Ratcliff and McKoon, 2008). Most tasks used in cognitive control studies (such as Simon task, flanker task and SART) require overriding or inhibiting a prepotent response, making these tasks very sensitive to the level of motor inhibition (Ridderinkhof, 2002; Ridderinkhof et al., 2004b; Dudschig and Jentzsch, 2009; van Driel et al., 2012). Errors in such tasks are mostly failures to inhibit fast automatic responses.

On the contrary, increased error RT is typically observed in complex or accuracy-demanding attentional tasks (Wilding, 1971; Luce, 1986; Dyson and Quinlan, 2003; Ratcliff and McKoon, 2008; O’Connell et al., 2009; Cohen and van Gaal, 2013). Slower RTs have been specifically associated with attentional lapses (Weissman et al., 2006; van Driel et al., 2012) and uncertainty (Pailing and Segalowitz, 2004; Wessel et al., 2011; Navarro-Cebrian et al., 2013). Apparently, decreased attention compromises stimulus encoding and subsequent task-specific processing, ultimately leading to the situation in which both correct and erroneous motor programs are almost equally activated, so that the response selection process takes longer time. Further, in the text, we will refer to this situation as “decision uncertainty”.

Importantly, correct responses may also differ in nature following a dichotomy similar to that known for erroneous responses. For example, some of the correct responses may in fact be preceded by subthreshold attempts to commit an erroneous response, resulting in increased RT on such “mixed correct” trials compared with “pure correct” trials (Cohen and van Gaal, 2014)—thus, again, providing evidence that decision uncertainty leads to delayed behavioral responses.

Performance monitoring, including error detection, is an essential component of the cognitive control framework. Importantly, error detection may be either internal (driven by endogenous processes) or external (driven by a feedback stimulus; Holroyd et al., 2004). Internal error detection is likely to occur in such conditions that information about perceived stimulus and task-specific stimulus-to-response mapping is of sufficient quality, so that information processing continuing beyond the moment of response initiation can result in an internal inference that another response should have been selected. On the contrary, after an erroneous response performed in the state of high decision uncertainty, there is not enough information to find out whether the response committed was correct or not; thus, only an external error detection is possible in this case—driven by an external feedback stimulus (Holroyd et al., 2004). We will refer to the latter situation as “outcome uncertainty”. Importantly, the same logic can be applied not only to error detection but also to correctness detection.

Outcome detection and ensuing cognitive control adjustments can be reflected in electroencephalographic (EEG) oscillations. A negative trial outcome (including both response-related internal error detection and feedback-related external error detection) is known to evoke increased frontal midline theta (FMT) oscillations in respective time windows (Yeung et al., 2004; Cohen et al., 2007, 2009; Cavanagh et al., 2009; Christie and Tata, 2009; Kolev et al., 2009; van de Vijver et al., 2011; Cavanagh and Frank, 2014; Novikov et al., 2015a). Suppression of alpha oscillations over posterior cortical areas presumably reflects adjustments of attention in attentional tasks (Carp and Compton, 2009; Mazaheri et al., 2009; van Driel et al., 2012) as well as an attentional enhancement during feedback expectation (Bastiaansen et al., 1999, 2002; Pornpattananangkul and Nusslock, 2016). Prefrontal activation in beta range can be observed in response to a positive feedback, supposedly signaling the importance of maintaining currently active task rules (van de Vijver et al., 2011).

The two types of errors viewed above may occur intermixed (van Driel et al., 2012). The two types of correct responses also go intermixed within an experiment (Cohen and van Gaal, 2014). Since RT differs between the types of responses outlined above, we expected that RT might be a valid approximation distinguishing trials with high and low levels of uncertainty caused by spontaneous lapses of attention.

The body of literature cited above suggests that fast errors are mostly premature responses resulting from failures to keep a sufficient motor threshold, leading to a state of low outcome uncertainty, which allows internal error detection soon after the response commission (i.e., before the feedback signal, if feedback is presented with a sufficient delay after the response). In such conditions, an external feedback error signal may be less informative—because it is predictable. On the contrary, slow trials are supposedly executed under a low level of sustained attention and the ensuing state of high decision uncertainty. Thus, slow errors mostly result from compromised task-specific processing. Consequently, on such trials, responses are followed by a state of high outcome uncertainty, so evaluation of trial outcome should mostly depend upon an external feedback signal—with negative feedback providing external error detection, and positive feedback providing an external reinforcing signal.

Thus, we predicted stronger error-related FMT on fast-RT trials (presumably, reflecting internal error detection) and stronger feedback-related FMT on slow-RT trials (presumably, reflecting external error detection). Since slow trials may be associated with lowered attentional level (Weissman et al., 2006; van Driel et al., 2012), we expected greater posterior alpha suppression specifically on erroneous slow trials—related to adaptive reconfiguration of attention and attentional enhancements related to feedback expectation in conditions of greatest outcome uncertainty. Additionally, we predicted that only on slow trials an increase in prefrontal beta oscillations in response to a positive feedback would be observed (van de Vijver et al., 2011; Cunillera et al., 2012).

In the current study, we focused on the within-subject analysis with distinction between fast and slow behavioral responses (relative to individual RT medians). We used an auditory version of the condensation task (Posner, 1964; Garner, 1974; Gottwald and Garner, 1975). This task is highly demanding for sustained attention while in contrast with many tasks used within the cognitive control paradigm (such as the Simon task, flanker task and SART) it involves no apparent inhibition of prepotent responses. It is characterized by substantially longer RTs compared to the Simon task, flanker task and SART, hinting at higher cognitive processing load needed to follow the complex conjunction-based stimulus-to-response mapping implemented within this task, making this task difficult to perform (Posner, 1964; Garner, 1974; Gottwald and Garner, 1975). Additionally, difficulty of the task may be expected to increase the importance of feedback signals, since even after extensive learning participants’ performance accuracy reaches a plateau of 85%–90% and does not improve any further (Lazarev et al., 2014). Both types of errors as defined by van Driel et al. (2012) can presumably be observed during the condensation task (Novikov et al., 2015a,b). Although ranges of correct and erroneous RTs substantially overlap, average RTs on erroneous trials are greater than average RTs on correct trials, thus hinting that a substantial fraction of errors is committed due to attentional lapses (Weissman et al., 2006; van Driel et al., 2012) and uncertainty (Pailing and Segalowitz, 2004; Wessel et al., 2011; Navarro-Cebrian et al., 2013). Thus, an attentional nature of the task specifically provides tools to compare trials with high and low levels of attention. The condensation task proved to be a valid experimental tool for studying EEG oscillations in the framework of cognitive control paradigm (Novikov et al., 2015a). In the current study, feedback was given with a sufficient delay after response commission, which allowed measuring both error-related and feedback-related brain events.

We found that error-related FMT was evident only on fast trials. We did not find the expected difference in the feedback-related FMT between slow and fast trials. Late post-response posterior alpha suppression was strongest on erroneous slow trials. Feedback-related prefrontal beta was present only on slow correct trials. Thus, the evidence obtained generally suggests that RT allows distinguishing the two types of trials, with fast trials related to higher levels of attention and low uncertainty, and slow trials related to lower levels of attention and higher uncertainty.

## Materials and Methods

### Participants and Experimental Conditions

Forty-nine healthy right-handed volunteers participated in the present study; their mean age was 23.5 ± 0.5 years (mean ± standard deviation), 31 females, and 18 males. All volunteers had normal or corrected-to-normal vision and normal hearing; they reported no history of auditory, neurological, or mental disorders. The experiments were carried out in accordance with the Declaration of Helsinki and its amendments and were approved by the ethics committee of the National Research University “Higher School of Economics (HSE)”. Informed consent was signed by each participant before the experiment. All experiments were conducted in a sound-attenuated chamber.

### Stimuli

Auditory stimuli were presented using E-Prime software (Psychology Software Tools, Inc., Sharpsburg, PA, USA) through a high-quality stereo headset with in-ear design at a sound pressure level of 90 dB (the sound pressure level at participants’ tympanic membrane could be a little lower depending on individuals’ external acoustic meatus anatomy that could prevent ideal sealing of the earpiece against the external acoustic meatus walls).

**Figure 1:**
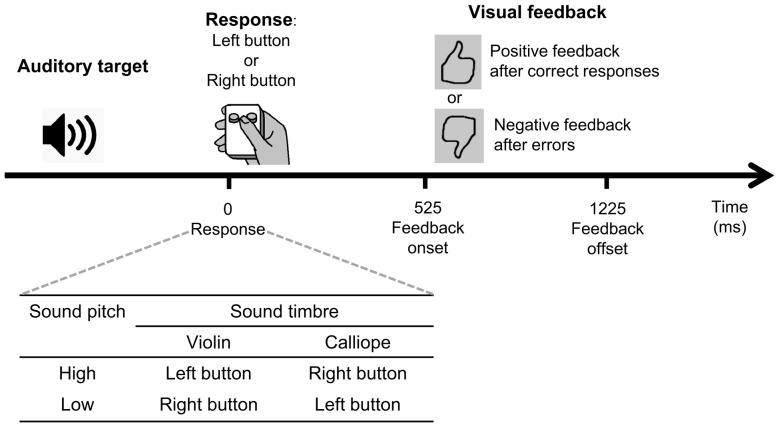
**A schematic illustration of the experimental behavioral task.** See text for details.

We used four pre-recorded auditory stimuli that were characterized by one of the two timbers (“violin” or “calliope”) and one of the two pitches (“low” 440 Hz, A4, or “high” 523.25 Hz, C5). The four stimuli were named in the instruction presented to the participants as “violin low”, “calliope low”, “violin high”, and “calliope high” (Figure 1, insert).

The tones were synthesized using Microsoft “GS Wavetable SW Synth” integrated into Microsoft DirectX (Microsoft Corporation, Redmond, WA, USA). For each tone, only a stationary plateau part was taken from the original digital recordings of sufficient length. The resulting duration of all auditory stimuli was 100 ms. Artificial rise and fall periods (each 10 ms in duration) were created by linearly decreasing amplitude represented in dB scale in a rising and falling fashion respectively. Mean square amplitudes of all auditory stimuli recordings were digitally equalized. Digital sound editing was done using Anvil Studio (Willow Software, Lake Forest Park, WA, USA), Audacity (Free Software Foundation, Boston, MA, USA), and MATLAB (MathWorks Inc., Natick, MA, USA).

Visual feedback stimuli were used: a positive visual feedback was a large black contour thumbs-up sign on a gray background, and a negative visual feedback was a thumbs-down sign, which was produced by rotating the thumbs-up sign by 180°(Figure 1).

### Design and Procedure

An auditory two-choice version of the condensation task (Posner, 1964; Garner, 1974; Gottwald and Garner, 1975) was used; a similar task was successfully employed in previous EEG studies of attention and cognitive control (Chernyshev et al., 2015; Novikov et al., 2015a).

The experiment included six identical blocks; after the end of each block, participants were offered a short rest. During each of the blocks, 100 auditory stimuli were presented; the four stimuli were presented with equal probabilities (25:25:25:25) interleaved in a quasi-random order, with random stimulus onset asynchrony (SOA) of 4000 ± 500 ms (uniform distribution).

Participants made responses to stimuli by pressing one or the other of the two specified buttons of a small hand-held gamepad with their thumb (Figure 1); they were instructed to hold the gamepad in their dominant (right) hand.

A schematic representation of a trial, as well as the stimulus-to-response mapping table are shown in Figure 1. The stimulus-to-response mapping table specifies the conjunction contingencies between the two stimulus features (“violin”/“calliope” and “high”/“low”) comprising the set of the four stimuli, and the response required to the left and right buttons of the gamepad. The nature of the condensation task is such that it cannot be performed successfully via processing any single feature alone: it requires a mental conjunction of two features dimensions (Posner, 1964; Garner, 1974; Gottwald and Garner, 1975).

Visual feedback was presented in all six experimental blocks, 525 ms after participants’ responses; duration of the feedback stimulus was 700 ms. After correct responses, participants were presented with a positive visual feedback; after erroneous responses they were presented with a negative visual feedback (Figure 1). The monitor screen was filled with a uniform gray background continuously between feedback presentations.

Feedback was presented only after responses with RTs longer than 300 ms. If RT exceeded 1700 ms, feedback stimuli were additionally supplemented with a word “Faster” on the monitor screen; both types of trials (“urged” responses and trials with abnormal RTs) were later excluded from the EEG analysis, since feedback was absent or modified on such trials compared with regular trials.

The participants were offered to familiarize with a table similar to that included in Figure 1 (insert), which was given to them printed out in a large font on a sheet of paper for free viewing, and then removed before the start of EEG recording. Before the start of the experimental blocks, the participants were also familiarized with the auditory stimuli: the experimenter manually presented them to the participants and named them orally, and then the participants were blind-tested with the stimuli. During this test, all of the participants easily named all of the stimuli correctly, and all of them stated confidently that they could clearly feel the difference between all of the stimuli and knew which button corresponded to each stimulus. The meaning of the feedback stimuli was explained in the instruction given to the participants before the start of the experiment.

The instruction also informed the participants that they were to press one of the two buttons as specified in the table, but it did not tell them to respond as fast as possible, nor did it force them to make random choices if they were not sure which response was correct.

### Behavioral Data Analysis

The first experimental block was intended for participants to get used to the task; it was demonstrated that under a similar condensation task participants reach plateau performance accuracy within the first experimental block (Lazarev et al., 2014). We included only the data from blocks 2–6 into all the analyses reported here.

At this stage, responses with RT shorter than 300 ms were excluded from the analysis because: (1) feedback was not given after such early responses; and (2) considering very large RTs characteristic of the condensation task (Lazarev et al., 2014; Novikov et al., 2015a,b), at least part of such responses could be in fact delayed responses belonging to the previous trial rather than to the current trial. Such responses were very rare (<0.3% of trials).

We calculated percentages of response types and average RTs for each subject using all corresponding trials (in the rare events of multiple button presses, only the first button press was accounted for). Two participants, who performed at lower than 50% level of accuracy, were excluded from this and all of the following analyses.

For each participant, median RT was calculated for all responses pooled together; this measure was used in all further analyses to account for the response speed.

All behavioral data analyses were performed within MATLAB (MathWorks Inc., Natick, MA, USA) using custom-made scripts. Comparisons were made using two-tailed paired *t*-tests.

### Electrophysiological Recording and EEG Preprocessing

The EEG was recorded using an NVX-52 system (Medical Computer Systems, Moscow, Russia) with Neocortex Pro software (Neurobotics, Moscow, Russia) from 27 electrodes in accordance with the modified international 10%-10% system and one electrooculogram electrode, with a linked earlobe reference. The band-pass filter was 0.1–200 Hz, and sampling rate was 1000 Hz. Electrode-to-skin impedance was kept below 10 kΩ for all channels.

EEG analysis was performed within MATLAB (MathWorks Inc., Natick, MA, USA) using custom-written scripts and built-in functions of EEGLAB toolbox (Delorme and Makeig, 2004). High-amplitude artifacts exceeding 300 μV were rejected from the data. Signals in bad channels were replaced by spherical interpolations over the neighborhood electrodes. Independent component analysis (ICA) was performed, and components related to eye movements were manually selected and rejected from the data. Finally, we substituted signals in channels contaminated with EMG by spherical interpolation over the neighborhood electrodes; we selected for this procedure those channels, in which the spectral power in 25–45 Hz range exceeded 1.5 standard deviations above the mean value taken over the total number of channels × blocks × subjects in the experimental sample (about 2% of channels × blocks × subjects).

In order to reduce volume conduction effects, current source density (CSD) transformation was applied to EEG data using open-source CSD toolbox (Kayser and Tenke, 2006a). CSD transformation can be applied to low-resolution EEG data (Kayser and Tenke, 2006b).

Response-locked epochs for each condition were extracted from the data (−2000 to 2000 ms relative to the response). Epochs were included into the EEG analysis only if they met the following conditions:

1. The RT was within 300–1700 ms range. Thus, “urged” responses and trials with abnormal RTs were excluded from the EEG analysis;
2. A single button press was committed during the trial. Thus, we excluded trials with multiple correcting responses that were occasionally performed by some participants and that could contaminate post-response EEG data;
3. The trial was preceded and succeeded by correct trials. Thus, only correct trials committed within sequences of correct trials and only single errors committed between correct trials were included into analysis. This was done in order to exclude post-error and pre-error effects influencing the trials that immediately follow or immediately precede erroneous responses.

### Classifying Trials into Four Experimental Conditions

Responses were classified as correct ones (a correct button was pressed) and errors (the other button was pressed). For each participant individually, median RT was calculated, and trials were additionally classified as “slow” or “fast” if RT was greater or shorter than the individual RT median correspondingly. Thus, four conditions were used in the further statistical analyses: slow correct, slow erroneous, fast correct and fast erroneous trials.

Since all participants committed less erroneous responses than correct responses, for the following EEG analysis we did a trial number matching procedure that equalized numbers of trials across conditions. This was needed to equalize the variance of mean non-phase-locked power estimate, thus avoiding the potentially huge bias in estimation of the mean difference in non-phase-locked power between conditions.

The trial number matching procedure was applied across all the four conditions specified above, independently within each participant’s dataset. All valid trials were retained within the least frequent condition, and the number of trials for other conditions was reduced to this number. For correct vs. erroneous contrasts (slow correct vs. slow erroneous, and fast correct vs. fast erroneous trials), trial number matching procedure involved an RT-matching procedure as follows. We used all trials from a condition that was less frequent throughout the experiment (erroneous trials), and for each of these trials we selected a matching trial from the other condition (correct trials) with the closest RT (each trial could be taken only once). This procedure equalized mean RTs within each pair under comparison, thus allowing us to compare correct trials with erroneous trials on compatible timelines. For slow vs. fast contrasts (slow correct vs. fast correct, and slow erroneous vs. fast erroneous trials), we excluded random trials from conditions with greater numbers of trials. Thus, within each individual participants’ dataset we produced a data subset with equalized numbers of trials across all four conditions (which was necessarily the smallest number across the four conditions).

After all the steps described above, we included into the further analyses only the EEG datasets that contained no less than five artifact-free trials in each of the conditions. Thus, all of the EEG analysis reported here was performed in 26 participants. For each participant each condition included 12.2 ± 5.8 trials (mean ± standard deviation), the range of individual values being 5–25 trials.

### Time-Frequency Decomposition

CSD signal in each channel was translated into time-frequency domain using wavelet transformation using sliding time windows at 20 ms steps. We used complex Morlet wavelets with the frequencies ranging from 2 Hz to 40 Hz in steps of 1 Hz; the number of cycles was linearly increased from 2 (on the lowest frequency) to 37.5 (on the highest frequency), thus providing an equal trade-off between time and frequency resolutions over the whole frequency range.

For each time-frequency bin and each electrode, we calculated non-phase-locked spectral power averaged over subsets of trials used for the analysis. First, we calculated the mean total power by averaging squared norms of complex amplitudes over the trials. Next, we calculated phase-locked power by averaging complex-valued amplitudes over the trials, and then taking squared norm of this sum. Non-phase-locked power was calculated as the difference between the total power and the phase-locked power.

Finally, we performed baseline normalization of non-phase-locked power, thus obtaining event-related spectral perturbation (ERSP). Baseline was calculated as an averaged spectral power over the −500 to 0 ms pre-stimulus time window (separately for each location and each frequency). We used a common baseline for the four conditions under comparison because we aimed to focus on post-stimulus effects and to get rid of possible pre-stimulus variation effects. The normalization was performed using the formula:

$$10\;\log_{10}\;(\text{power/baseline}),$$

so that the event-related changes in non-phase-locked spectral power relative to baseline were expressed in decibels.

### ROI Definition

The current study primarily aimed at testing specific predictions concerning error-related and feedback-related events. Thus, we defined the following *a priori* ROIs based on previous research.

ROI 1—error-related and feedback-related FMT oscillations (Yeung et al., 2004; Cohen et al., 2007, 2009; Cavanagh et al., 2009; Christie and Tata, 2009; van de Vijver et al., 2011; Cavanagh and Frank, 2014; Novikov et al., 2015a): 4–7 Hz, frontal midline electrodes centered on Fcz (Fz, Fcz, Cz), 100–300 ms after response and 200–500 ms after feedback onset correspondingly.

ROI 2—posterior alpha oscillations related to error-related attentional reconfiguration (Klimesch, 1999; Carp and Compton, 2009; Mazaheri et al., 2009; van Driel et al., 2012; Novikov et al., 2015a) and to attentional enhancement during feedback expectation (Bastiaansen et al., 1999, 2002; Pornpattananangkul and Nusslock, 2016): 8–13 Hz, parieto-occipital electrodes (Pz, O1, Oz, O2), 400–700 ms after response.

ROI 3—feedback-related frontal beta oscillations (Cohen et al., 2007; Marco-Pallares et al., 2008; van de Vijver et al., 2011; Cunillera et al., 2012): 15–25 Hz, electrodes overlaying prefrontal areas (F3, Fz, F4), 200–500 ms after feedback onset.

### Statistical EEG Data Analysis and Illustrations

Within each ROI, ERSP values were averaged across locations, time windows and frequency ranges specified above. We used a two-factor analysis of variance (ANOVA) with repeated measures, with factors speed (two levels: slow and fast) and accuracy (two levels: correct and erroneous). *Post hoc* comparisons were made with Fisher’s least significant difference (LSD) test.

In order to illustrate validity of our selection of frequency bands and time windows, we did additional analyses within spatial regions corresponding to ROIs specified above. In these analyses, we explored timecourses, as well as time-frequency patterns of the effects under study. We used two types of paired comparisons in these analyses. First, for each condition, we compared ERSP values with zero, thus testing whether activity in temporal or time-frequency bins differed from the respective baseline values. Second, we compared ERSP values between correct trials and erroneous trials (to show whether activity depended upon trial outcome); this was done separately for slow and fast subsets of trials.

For each of the paired comparisons described above, we applied permutational statistical testing based on the threshold-free cluster enhancement (TFCE; Smith and Nichols, 2009). First, we calculated paired *t*-statistics for each data bin, thus producing a map of *t-scores* (one-dimensional for timecourse analyses and two-dimensional for time-frequency analyses). Next, we applied to this map the TFCE algorithm. After that, we shuffled the initial data (by reversing the sign of ERSP values in the analyses aiming at comparing the activity of interest vs. baseline, or by flipping condition labels in the cross-condition analyses) for random subset of subjects. In both cases, this was done for all data bins within a participant’s dataset simultaneously, so the inherent dependence of adjacent data bins was not broken by the shuffling procedure. Next, we calculated the TFCE-transformed map on the shuffled data; this was repeated 1000 times. Positive and negative *t-scores* were transformed to TFCE scores using two independent runs of algorithm. At each permutation step, we obtained the maximal (positive) and the minimal (negative) TFCE-score over the entire map, and then we constructed two distributions: one for the maximal and the other for the minimal values. Finally, for each bin of the non-shuffled TFCE matrix (independently), we calculated the quantiles of “minimal” and “maximal” distributions the value in this bin falls into, thus obtaining permutation-based *p-value* for this bin. All results reported here were considered significant at *p* < 0.05. In order to improve signal-to-noise ratio, we did this analysis after averaging data within each of the consecutive five time points using a rectangular time window, thus increasing the step of data representation from 20 ms to 100 ms.

## Results

### Behavioral Data

Two participants were excluded from the analysis due to insufficient behavioral performance, and 21 participants were excluded from the analysis due to insufficient number of valid erroneous trials remaining after the rigorous procedures applied as described above. Thus, the results reported here include 26 participants. These participants made on average 76.3 ± 12.5% (mean ± standard deviation) of correct responses, and 22.9 ± 12.1% of errors. Average RT was 1082 ± 173 ms on correct trials, and 1164 ± 225 ms on erroneous trials. RT on erroneous trials was significantly larger than on correct trials (*t*_(25)_ = −83.1, *p* < 0.001).

Average overall individual median RT was 1096 ± 195 ms; this measure was used to classify responses into slow and fast trials on within-subject basis.

### FMT Oscillations at Mid-Frontal ROI 1

**Figure 2:**
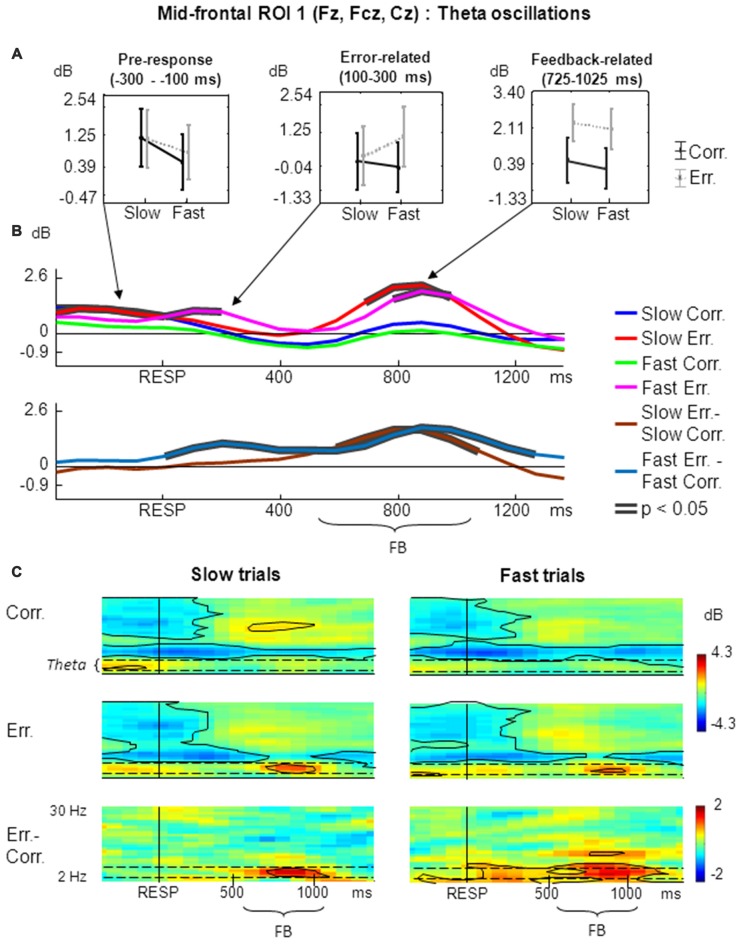
**Non-phase-locked frontal midline theta (FMT) oscillatory activity at mid-frontal ROI 1 on fast and slow trials. (A)** Graphs representing averaged event-related spectral perturbation (ERSP) data on slow correct, slow erroneous, fast correct and fast erroneous trials. Panels represent “pre-response”, “error-related” and “feedback-related” time windows correspondingly; data are plotted as mean ± standard error of mean. **(B)** Timecourses of ERSP modulations relative to response. (Top subpanel: slow correct, slow erroneous, fast correct and fast erroneous trials. Bottom subpanel: difference between slow erroneous and slow correct, and between fast erroneous and fast correct trials). Black contours overlaid on timecourse lines indicate statistical significance (*p* < 0.05, permutation statistics). “RESP”—behavioral response, “FB”—feedback. Time is shown relative to the behavioral response. **(C)** Time-frequency plots of oscillatory activity relative to response. Left panels: ERSP distribution on slow trials. Right panels: ERSP distribution on fast trials. Horizontal dashed lines over ERSP plots indicate theta frequency range. Within each panel: “Corr.”: dynamics of ERSP on correct trials; “Err.”: dynamics of ERSP on erroneous trials; “Err.–Corr.”: dynamics of ERSP difference between erroneous and correct trials. Black contours show significant time-frequency areas (*p* < 0.05, permutation statistics). “RESP”—behavioral response, “FB”—feedback. Time is shown relative to the behavioral response.

The results of non-phase-locked FMT oscillations analysis within frontal midline ROI 1 are shown in Figure 2.

For the non-phase-locked theta power averaged across 100–300 ms post-RT window, the main effect of accuracy was significant (*F*_(1,25)_ = 4.97, *p* = 0.04, partial *η*^2^ = 0.166). There was also a significant interaction between factors speed and accuracy (*F*_(1,25)_ = 5.20, *p* = 0.03, partial *η*^2^ = 0.172). *Post hoc* tests revealed that theta power was significantly higher on fast erroneous trials compared with slow erroneous, slow correct and fast correct trials (*p* < 0.05; Figure 2A, middle panel).

Analysis of non-phase-locked theta power averaged across 200–500 ms after feedback onset (725–1025 ms after the behavioral response) revealed that the main effect of accuracy was highly significant (*F*_(1,25)_ = 69.33, *p* << 0.001, partial *η*^2^ = 0.735). The main effect of speed and interaction between factors speed and accuracy were not significant (*p* > 0.05). *Post hoc* analysis revealed that theta power was significantly higher on slow erroneous trials compared with slow correct and fast correct trials, and on fast erroneous trials compared with slow correct and fast correct trials (*p* < 0.001; Figure 2A, right panel).

Figure 2B represents timecourses of non-phase-locked theta power. The error-related and feedback-related effects described above are evident in the timecourses. Timecourses also reveal that feedback-related theta on slow erroneous trials had an apparently earlier and stronger onset. After the response, differential effect was significant on fast trials, while during the feedback the differential effect was significant both on slow- and fast trials.

Figure 2C illustrates corresponding time-frequency plots. Inspection of these figures confirms that error-related theta activity was statistically significant only on fast trials (Figure 2C, right panels) but not on slow trials (Figure 2C, left panels). Feedback-related theta was present both on slow and fast trials (Figure 2C, left and right panels).

Visual inspection of timecourses and time-frequency plots suggests that before commission of a response theta activity was higher on slow trials. Although it was not the main focus of this study, we applied ANOVA to the theta power averaged over the pre-RT window of −300 to −100 ms, and found the main factor speed to be highly significant (*F*_(1,25)_ = 13.12, *p* = 0.001, partial *η*^2^ = 0.344; Figure 2A, left panel).

### Alpha Oscillations at Posterior ROI 2

**Figure 3:**
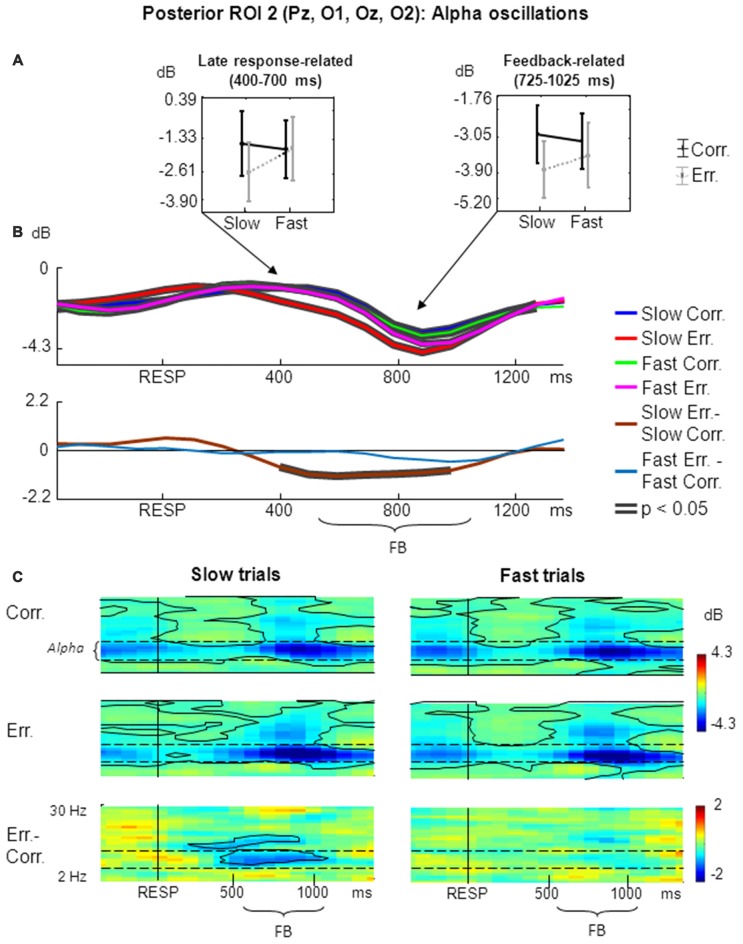
**Non-phase-locked alpha oscillatory activity at posterior ROI 2 on fast and slow trials. (A)** Graphs representing averaged ERSP data on slow correct, slow erroneous, fast correct and fast erroneous trials. Panels represent “late post-response” and “feedback-related” time windows correspondingly; data are plotted as mean ± standard error of mean. **(B)** Timecourses of ERSP modulations relative to response. Conventions as in Figure 2; **(C)** Time-frequency plots of oscillatory activity relative to response. Horizontal dashed lines over ERSP plots indicate alpha frequency range. Other conventions as in Figure 2.

The results of non-phase-locked alpha oscillations analysis within posterior ROI 2 are shown in Figure 3.

For the non-phase-locked alpha power averaged across 400–700 ms post-RT window, the main effects of speed and accuracy where both non-significant (*p* > 0.05). Yet interaction between factors speed and accuracy was significant (*F*_(1,25)_ = 4.51, *p* = 0.04, partial *η*^2^ = 0.153). *Post hoc* comparisons revealed that alpha power was significantly lower on slow erroneous trials compared with fast erroneous trials and slow correct trials (*p* < 0.05; Figure 3A, left panel).

Figure 3B represents timecourses of non-phase-locked alpha theta power. Visual inspection of the timecourses confirms that during a late post-response and early-feedback-related time alpha suppression was stronger on slow erroneous trials compared with other conditions. Differential effect was significant only for slow trials.

Figure 3C illustrates corresponding time-frequency plots. As can be seen in the timecourses, alpha suppression was stronger on slow erroneous trials than on other types of trials within a prolonged time window starting from approximately 300–400 ms and ending around 900–1000 ms.

Time-frequency plots also confirm that that there was a significant difference in alpha power between slow erroneous and slow correct trials starting about 400 ms and extending towards the end of the analysis period (Figure 3C, left panels), while no such difference existed on fast trials (Figure 3C, right panels).

Visual inspection of time courses and time-frequency plots shows that, during a later part of feedback period alpha power was stronger suppressed on erroneous trials than on correct trials—both for slow and fast responses. Although this was not the primary aim of the current study, we applied ANOVA to the alpha power averaged within the feedback time window of 200–500 ms after the feedback onset (i.e., 725–1025 ms after the behavioral response). The main effect of accuracy was significant (*F*_(1,25)_ = 9.80, *p* = 0.004, partial *η*^2^ = 0.282; Figure 3A, right panel).

### Beta Oscillations at Frontal ROI 3

**Figure 4:**
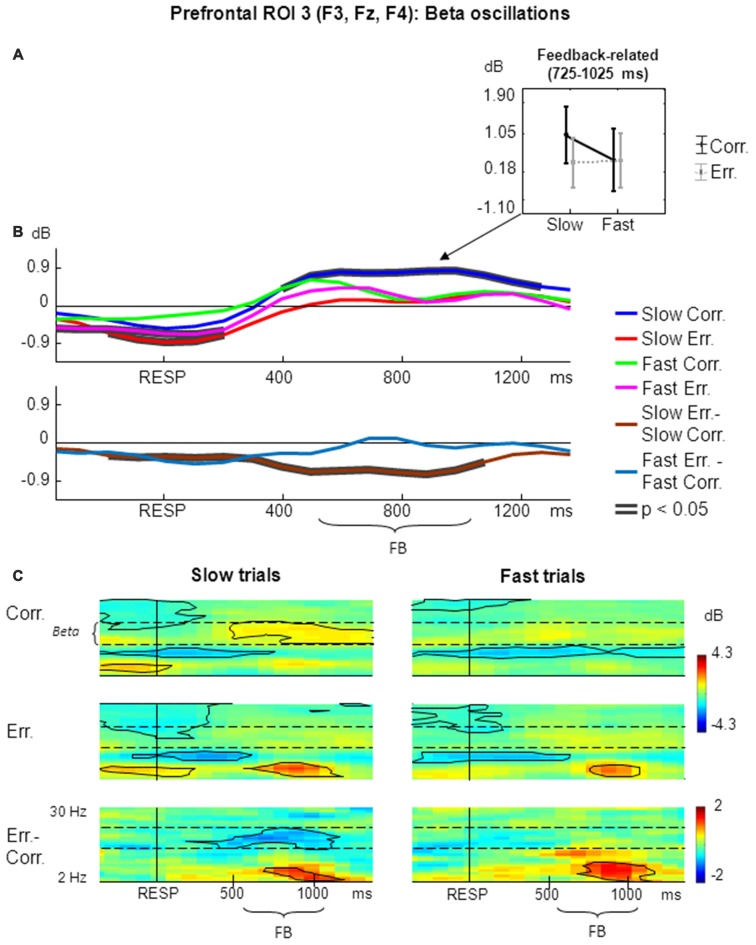
**Non-phase-locked beta oscillatory activity at prefrontal ROI 3 on fast and slow trials. (A)** Graphs representing averaged ERSP data on slow correct, slow erroneous, fast correct and fast erroneous trials. Panel represents feedback-related time window; data are plotted as mean ± standard error of mean. **(B)** Timecourses of ERSP modulations relative to response. Conventions as in Figure 2; **(C)** Time-frequency plots of oscillatory activity relative to response. Horizontal dashed lines over ERSP plots indicate beta frequency range. Other conventions as in Figure 2.

The results of non-phase-locked beta oscillations analysis within prefrontal ROI 3 are shown in Figure 4.

For the non-phase-locked beta power averaged across the feedback time window of 200–500 ms after the feedback onset (i.e., 725–1025 ms after the behavioral response), significant were the main effect of speed (*F*_(1,25)_ = 8.50, *p* = 0.007, partial *η*^2^ = 0.254), the main effect of accuracy (*F*_(1,25)_ = 5.29, *p* = 0.03, partial *η*^2^ = 0.175), as well as their interaction (*F*_(1,25)_ = 7.03, *p* = 0.01, partial *η*^2^ = 0.219). *Post hoc* analysis revealed that beta power was significantly higher on slow correct trials compared with fast correct trials, slow erroneous trials and fast erroneous trials (*p* ≤ 0.002; Figure 4A).

Figure 4B represents timecourses of non-phase-locked beta power. Visual inspection of the timecourses confirms that feedback-related beta activation was significantly stronger on slow correct trials compared with fast correct, slow erroneous, and fast erroneous trials. Differential effect was significant only for slow trials.

Figure 4C illustrates corresponding time-frequency plots. The effect of feedback-related beta activation can also be seen on time-frequency plots. On slow correct trials, there was a significant prolonged feedback-related activation in beta range that started approximately at the feedback onset and lasted to the end of the analysis period (Figure 4C, left panels). The difference between slow erroneous and slow correct trials also revealed a significant prolonged feedback-related effect in beta range that possibly started around the time of feedback onset and lasted to the end of the feedback presentation. In contrast, no such effects were present on fast trials (Figure 4C, right panels).

## Discussion

### Summary of Results

In the present study, we examined the dynamics of oscillatory activity in theta, alpha and beta bands in relation to correct and erroneous behavioral responses during performance of the auditory condensation task. We focused on the distinction between fast and slow behavioral responses.

In line with our predictions, we found that error-related FMT was present only on fast trials, but not on slow trials. Yet we did not find a statistical evidence of the expected difference in the feedback-related FMT power between slow and fast trials. As we expected, late post-response posterior alpha suppression was strongest on erroneous slow trials. Also, in line with our predictions, feedback-related prefrontal beta was present only on slow correct trials.

### Behavioral Performance

The behavioral task used in the present study was sufficiently demanding on the participants: they committed on average 22.9% of errors. RT was very long in this task: 1082 ms on correct trials, and 1164 ms on erroneous trials. This clearly differs the condensation task from many behavioral tasks typically used within the cognitive control paradigm, such as Simon task, flanker task and SART (Ridderinkhof, 2002; Ridderinkhof et al., 2004a; Dudschig and Jentzsch, 2009; van Driel et al., 2012).

Average RTs in the present study were even longer, than in another version of the auditory condensation task used in our previous study (864 ms and 976 ms correspondingly; Novikov et al., 2015a). Percentage of errors was also higher in the present study (22.9%) than in the previous one (10.2%). Although the behavioral procedures were very similar between the two studies, in the present study we used a new set of auditory stimuli. Importantly, in the present study, the difference in pitch between “low” and “high” stimuli was smaller than in the previous one (yet it should be emphasized that it was about an order of magnitude above a typical differential pitch threshold). Supposedly, the task in the present study imposed even higher attentional load on the participants, than the one in our previous study.

In the present study, RTs of erroneous responses were significantly longer than correct RTs. Error slowing is typically observed in complex attentional tasks (Wilding, 1971; Luce, 1986; Dyson and Quinlan, 2003; Ratcliff and McKoon, 2008; O’Connell et al., 2009; Cohen and van Gaal, 2013). Moreover, error slowing is characteristic of attentional lapses (Weissman et al., 2006; van Driel et al., 2012) and of conditions of uncertainty (Pailing and Segalowitz, 2004; Wessel et al., 2011; Navarro-Cebrian et al., 2013). On the contrary, error speeding is typical of tasks that depend upon proper regulation of motor threshold (Ridderinkhof, 2002; Ratcliff and McKoon, 2008; Dudschig and Jentzsch, 2009), and errors in such tasks mostly result from inappropriate action impulses, that could not be blocked by an insufficiently low motor threshold (van Driel et al., 2012).

We can conclude that behavioral data obtained show that the task used in the present study was attentionally demanding. Thus, unlike the tasks with strong dependence on motor threshold, the current task allowed us to study errors related to attention.

### FMT Oscillations

As can be seen in Figure 2, FMT power was elevated before the response commission. Such activity is believed to reflect integration of task-related processes (stimulus processing, memory encoding and retrieval, maintenance of task rule representations, activation of motor programs, etc.) through top-down control mechanisms, as well as conflict detection and resolution; FMT was shown to have sources in the medial prefrontal cortex (Womelsdorf et al., 2010; Cavanagh and Frank, 2014).

In the pre-RT window, FMT activity was significantly stronger on slow trials compared with fast ones. Slow responses are supposedly committed in the state of high decision uncertainty, when the need to choose one correct response between the two competing yet almost equally activated motor programs supposedly invokes stronger activation of task-related neural processes and their coordination with cognitive control mechanisms; this set of brain processes is reflected in stronger FMT activation (Cavanagh et al., 2009; Cavanagh and Frank, 2014). On the contrary, fast responses are supposedly committed in the state of low decision uncertainty (i.e., they involve no prolonged competition between motor programs, since one of the programs quickly surpasses the low motor threshold), thus no such strong activation of processes related to cognitive control occurs before the response. This is compatible with our assumption that response speed may be related to the level of decision uncertainty (Wilding, 1971; Luce, 1986; Dyson and Quinlan, 2003; Ratcliff and McKoon, 2008; O’Connell et al., 2009; Cohen and van Gaal, 2013).

In the post-RT window, we observed enhanced theta power after erroneous responses compared to correct responses (the main effect of accuracy was significant). This effect was repeatedly replicated in literature and it is thought to reflect error detection process (Luu and Tucker, 2001; van Driel et al., 2012; Navarro-Cebrian et al., 2013; Novikov et al., 2015a). Analysis of EEG oscillations in relation to error-related negativity revealed that theta oscillations may be associated with error detection at the level of movement monitoring; the effect was mostly pronounced for the total theta power rather than for phase-locked theta power, thus hinting that an increase in theta power rather than in synchronization (phase-locking) is responsible for this effect (Yordanova et al., 2004; Kolev et al., 2009). Multiple studies reported that theta increase after incorrect responses originates in the medial prefrontal cortex and acts as a signal of need for increasing the level of cognitive control (Ridderinkhof et al., 2004a; Debener et al., 2005; Cohen et al., 2008; Doñamayor et al., 2012). Moreover, Cavanagh and Frank (2014) suggested that enhanced theta synchronization between the medial prefrontal cortex and other parts of the brain after error commission reflects implementation of higher cognitive control for regulation of future actions.

As we expected, we found a statistically significant interaction between speed and accuracy during a post-RT window: theta power was significantly higher after fast erroneous responses compared with other conditions, supporting the hypothesis that the two types of incorrect responses differ as far as the internal error detection concerns. Fast errors were followed by an increase in FMT that could indicate internal error detection. On the contrary, the effect was totally absent after slow erroneous responses. Therefore, internal error detection occurs only after fast errors, which are followed by the state of low outcome uncertainty, and it is absent after slow errors, which are followed by the state of higher outcome uncertainty.

This logic is compatible with the notion that two different types of errors may occur due to disruption of two different processes involved in successful response completion, namely failures of motor control and attentional lapses (van Driel et al., 2012). Likewise, the study of Navarro-Cebrian et al. (2013) demonstrated enhanced theta power after aware errors (which were committed faster than correct responses) and no such enhancement after responses with self-reported outcome uncertainty (which were committed much slower than all other responses).

During the feedback time window, FMT activity was significantly stronger on erroneous trials compared with correct ones. Generally, such an effect is considered to reflect detection of a mismatch between an expected and an actual outcome (Cohen et al., 2007, 2009; Cavanagh et al., 2010; van de Vijver et al., 2011). The feedback-related FMT effect can be viewed as an external error detection leading to an adaptive increase in cognitive control. Thus, in line with our previous report using a different version of the condensation task (Novikov et al., 2015a), the data obtained show that the auditory condensation task evokes the typical FMT effects, which were previously observed in substantially different behavioral tasks—such as Simon task, flanker task and SART (Ridderinkhof, 2002; Ridderinkhof et al., 2004a; Dudschig and Jentzsch, 2009; van Driel et al., 2012).

According to our predictions, the effect of feedback-related theta power activation should have been stronger on slow erroneous trials, because after fast errors the internal error detection may have already occurred before the feedback onset (525 ms in our experiment), making feedback less informative—as compared with the case of slow errors followed by the state of high outcome uncertainty. Indeed, visual inspection of the timecourses and time-frequency plots in Figures 2B,C reveals somewhat earlier and stronger FMT onset on slow erroneous trials compared with fast erroneous trials. However, according to ANOVA results, the feedback-related FMT did not statistically differ between slow and fast trials. Thus, regarding processes which FMT reflects, external feedback seems to be informative on both certain and uncertain erroneous trials. This may seem to contradict the report of Holroyd et al. (2004): yet the level of certainty under the task in that study could be higher compared with conditions of much less straightforward stimulus-to-response mapping of the condensation task used in our study. Additionally, since the task used in this study was quite challenging and required significant effort and motivation on the part of the participants, it is possible that negative feedback was always perceived by them as a salient negative signal—no matter whether it could be predicted internally on a particular trial or not.

### Posterior Alpha Oscillations

As can be seen in Figure 3, alpha power was significantly suppressed during post-response and feedback time windows. It is believed that alpha suppression is a sign of cortical disinhibition, which is required for a variety of cognitive processes recruited during performance of tasks demanding top-down control (Klimesch et al., 2007). Numerous studies link alpha suppression to an adaptive increase in attentional level required for optimal task performance (Carp and Compton, 2009; Compton et al., 2011; Cohen and Ridderinkhof, 2013).

We found a significant differential effect at a late post-RT interval, starting around 400 ms after the behavioral response and continuing beyond the feedback onset: depression of posterior alpha oscillations was stronger on slow erroneous trials compared with correct trials. Similar effects on erroneous trials were reported in previous studies, and they were interpreted as an adaptive enhancement of sustained attention resulting from error detection (Carp and Compton, 2009; Mazaheri et al., 2009; Novikov et al., 2015a). Thus, together with our previous report using a similar version of the condensation task (Novikov et al., 2015a), our results confirm effects obtained in other previous studies.

If alpha suppression is indeed caused by an internal detection of an error that has just been committed, one could expect that this effect would be stronger on fast erroneous trials, during which, according to our results, the internal error detection (accompanied by corresponding error-related theta oscillations) was much more evident than during slow erroneous trials. Yet our findings revealed an opposite pattern: we observed the most significant posterior alpha suppression specifically on slow erroneous trials. This observation does not seem to agree with the notion that error detection manifests itself as an increase in theta power (Luu and Tucker, 2001; Yeung et al., 2004; Cohen, 2011) and that post-error depression of alpha oscillations comes as a consequence of such an increase in theta power (Mazaheri et al., 2009). Thus, the mechanism of internal error detection is an unlikely explanation of the current finding.

According to our hypothesis, slow behavioral responses may be a consequence of lowered level of attention, while fast responses may be a consequence of lowered motor threshold. Speculatively, the current finding could be interpreted as an adaptive attentional enhancement that follows attentional lapses on slow trials, while on fast trials there is no need in such an attentional enhancement because attention is not compromised. Lowered level of attention is related to deterioration in specific task information processing, leading to decision uncertainty. Following this logic, we can hypothesize that either attentional lapses or the ensuing state of uncertainty may be detected, this detection leading to adaptive attentional reconfiguration. If this is true, the following question arises: why stronger alpha-band suppression occurs specifically on slow erroneous trials rather than on both types of slow trials.

As a tentative explanation, we can consider a continuum of attentional lapses of varying deepness. The deeper the loss of attention is, the greater the uncertainty is—both decision uncertainty and trial outcome uncertainty—due to compromised task-specific information processing. Thus, apparently, the likelihood to commit an error is greater. The deeper an attentional lapse is, the stronger influence on parietal cortex is needed to recover lost attention. Thus, on slow trials, there may be a tendency towards coincidence between increased chance of error commission and stronger signal for attentional reconfiguration (triggered by detection of an attentional lapse and/or a state of uncertainty). This probabilistic coincidence seems to be a likely explanation of the results obtained.

According to van Driel et al. (2012), adaptive processes invoked by attentional lapses themselves are related to the increases in post-error alpha desynchronization and are not related to theta power modulations. Thus, our results may confirm and extend the view of two separate task monitoring mechanisms reflected in two different oscillation bands—theta and alpha. Meanwhile, it is not clear, whether the increased alpha suppression observed in the current study reflects detection of attentional lapses, and/or uncertainty itself, and/or adaptive adjustments induced by this detection.

Since the alpha suppression effect apparently started before feedback onset and continued through the early period of feedback processing, one can propose a further refinement to the interpretation of the posterior alpha power modulations within this time interval. Since slow errors supposedly occur in conditions of greatest uncertainty, feedback signal is the only source of information to resolve the trial outcome. Thus, we can expect the enhancement of attention specifically related to anticipation of the visual feedback (in addition to a probable general increase in attention adaptively addressed to future trials). Indeed, feedback anticipation was linked to posterior alpha suppression in a number of reports (Bastiaansen et al., 1999, 2002; Pornpattananangkul and Nusslock, 2016). According to the same logic, alpha suppression after feedback onset may be related to enhanced attention during feedback processing. Alpha oscillations are known to be suppressed by feedback presentation (Papo et al., 2007; Luft et al., 2014), although the effect is usually manifested during a later period of feedback processing. The difference in timing of the effect may be explained by the nature of the condensation task that demands greater attentional involvement in comparison with many behavioral tasks commonly used, thus leading to earlier attention-related alpha suppression. Additionally, the anticipatory adaptive attentional enhancement occurring specifically on slow erroneous trials may lead to an increased processing of the visual feedback, thus also resulting in stronger alpha suppression in the posterior cortex (Pfurtscheller et al., 1994). Stimulus-related alpha depression in the occipital cortex is known to be stronger when the stimulus is attended compared with the ignored condition (Zumer et al., 2014). Thus, the effect observed may be a cumulative result of several related mechanisms, all of which seem to be a result of the process of detection of attentional lapses and/or outcome uncertainty.

Within the later time window related to feedback processing, alpha suppression manifested a different pattern: it was stronger for errors compared with correct responses. It seems that negative feedback leading to external error detection itself results in the enhancement of attention. Other studies also demonstrated an effect of negative feedback-related alpha suppression and interprete it as enhanced arousal, alertness and attention induced by external error detection (Papo et al., 2007; Jung et al., 2010; Luft et al., 2014).

### Frontal Beta Oscillations

Strong modulations of beta power over the prefrontal cortex (Figure 4) were clearly visible in the current study, which was based on the condensation task known to be cognitively demanding (Chernyshev et al., 2015). A number of previous reports demonstrated modulation of beta-band activity in the prefrontal areas during action planning (Siegel et al., 2011), implementation of cognitive control (Zhang et al., 2015), working memory load (Babiloni et al., 2004) and feedback processing (Cohen et al., 2007; Marco-Pallares et al., 2008; van de Vijver et al., 2011; Cunillera et al., 2012). Using combined EEG-fMRI analysis, Mas-Herrero et al. (2015) revealed that beta oscillations reflect involvement of frontal, striatal and hippocampal structures related to memory during reward processing.

In our study, we observed a significant increase in beta power on correct trials during presentation of the positive feedback stimulus over the prefrontal ROI, as previously reported by Cunillera et al. (2012). Our results generally stay in line with other reports that showed enhanced beta-band oscillations in the prefrontal cortex induced by positive feedback during reinforcement learning (Cohen et al., 2007; van de Vijver et al., 2011) and gambling tasks (Marco-Pallares et al., 2008).

We found that the effect of beta power increase clearly differs between fast and slow correct responses. On slow correct trials, the increase was prominent and statistically significant, lasting through the whole feedback presentation and beyond it. On the contrary, on fast correct trials, beta power slightly rose and then receded shortly after the feedback onset. The most significant increase in beta power was observed around 250 ms after feedback onset, and it lasted through the whole presentation period. Such modulations were observed exclusively for slow correct responses, making this condition stand apart from all other conditions.

We believe that slow responses are followed by the state of high outcome uncertainty, which precludes any immediate internal error detection, thus making the sign of the external feedback unexpected and informative it terms of trial outcome detection. Similarly, previous studies suggested that beta oscillations increase after processing of unexpected feedback as a response to feedback valence (Marco-Pallarés et al., 2015).

Moreover, the lack of immediate internal detection makes external feedback the only reliable source of information about the accuracy of the current trial. Therefore, feedback processing is crucial in order to optimize future behavior through learning mechanisms. In our study, enhanced beta oscillations after the unexpected positive feedback may reflect recruitment of reward processing and reinforcement mechanisms. This is compatible with a view proposed by van de Vijver et al. (2011) that beta-band oscillations might be responsible for the global synchronization of neural assemblies in order to strengthen the current response. Accordingly, we observed the most prominent beta oscillations after slow correct responses, which were committed under the state of high decision uncertainty and followed by high outcome uncertainty, making feedback necessary to detect whether response was correct, and, if so, to reinforce maintenance of current task rules. Likewise, Engel and Fries (2010) proposed that beta band modulations are associated with maintenance of the status quo, the ability to sustain the current cognitive state.

## Conclusion

We hypothesized that RT might be a valid approximation distinguishing trials that differ in the levels of sustained attention and decision uncertainty.

Fast errors, which are supposed to be premature responses (van Driel et al., 2012), can be detected internally, because information processing may continue to progress normally after the moment of motor program initiation—the effect, which is well described in literature in association with a corresponding FMT activation (Yeung et al., 2004; Cavanagh et al., 2009; Cavanagh and Frank, 2014). On the contrary, internal error detection is much less likely to occur after slow errors, which are related to attentional lapses (van Driel et al., 2012), supposedly due to outcome uncertainty, because specific processing of task-related information is compromised and there is not enough evidence to decide whether an error was made. The results obtained in the current study indeed provided evidence of error-related FMT oscillations only on fast trials, thus staying in line with our predictions.

We expected that feedback might be more informative in situations of higher outcome uncertainty after slow responses, when only an external feedback signal can provide proper trial outcome evaluation. In fact, we did not find a statistically compelling evidence that feedback-related FMT oscillations were stronger on slow trials than on fast trials. Yet we found a strong evidence of feedback-related prefrontal beta oscillations occurring only on slow trials but not on fast trials. Thus, in terms of mismatch between the actual and the expected outcomes, both trial types revealed strong processing of the negative feedback signal (Cohen et al., 2007, 2009; Cavanagh et al., 2010; van de Vijver et al., 2011)—revealing that the negative feedback was perceived as a salient negative signal even if it could be predicted internally. However, in terms of feedback being a positive reinforcer—as evidenced by frontal beta activation (Cohen et al., 2007; Marco-Pallares et al., 2008; van de Vijver et al., 2011; Cunillera et al., 2012), our findings stay well in agreement with our predictions.

Additionally, pre-response FMT oscillations were stronger before slow responses thus revealing stronger activations of brain processes related to cognitive control. This agrees with our assumption that response speed may be related to the level of decision uncertainty (Wilding, 1971; Luce, 1986; Dyson and Quinlan, 2003; Ratcliff and McKoon, 2008; O’Connell et al., 2009; Cohen and van Gaal, 2013). Late post-response posterior alpha power was most strongly reduced on slow erroneous trials, thus also staying in line with our predictions and confirming that slow responses are related to the states of decreased attention and higher decision and outcome uncertainty. These findings are in agreement with a large body of literature (Luu et al., 2004; Pailing and Segalowitz, 2004; Weissman et al., 2006; Wessel et al., 2011; van Driel et al., 2012; Navarro-Cebrian et al., 2013).

In summary, our findings demonstrate that slow and fast responses differ in the nature of cognitive phenomena involved such as performance monitoring, attention, decision uncertainty and reinforcement. Additionally, the current study demonstrates that using attentional tasks may be an effective tool for investigation into the nature of cognitive control and related phenomena.

## Author Contributions

NAN, EEK, AAL and BVC: conception or design of the work. YMN, NAZ and AAL: acquisition of data. NAN, YMN and EEK: analysis of data. NAN, YMN, NAZ and BVC: interpretation of data. YMN, NAZ and BVC: drafting the work. NAN, EEK and AAL: revising the work critically for important intellectual content. NAN, YMN, NAZ, EEK, AAL and BVC: final approval of the version to be published; agreement to be accountable for all aspects of the work in ensuring that questions related to the accuracy or integrity of any part of the work are appropriately investigated and resolved.

## Funding

All authors are supported by the Basic Research Program at the National Research University Higher School of Economics (HSE) in 2016.

## Conflict of Interest Statement

The authors declare that the research was conducted in the absence of any commercial or financial relationships that could be construed as a potential conflict of interest.
